# Blood pressure control and its determinants among diabetes mellitus co-morbid hypertensive patients at Jimma University medical center, South West Ethiopia

**DOI:** 10.1186/s40885-017-0085-x

**Published:** 2017-12-27

**Authors:** Sintayehu Muleta, Tsegaye Melaku, Legese Chelkeba, Desta Assefa

**Affiliations:** 1 Department of Clinical Pharmacy, School of Pharmacy, Institute of Health, Jimma University, P.O.Box:378, Jimma, Oromia Ethiopia; 2 Department of Pharmaceutics, School of Pharmacy, Institute of Health, Jimma University, P.O.Box:378, Jimma, Oromia Ethiopia

**Keywords:** Hypertension, Diabetes mellitus, Blood pressure control, Uncontrolled hypertension

## Abstract

**Background:**

Hypertension is the major contributor to cardiovascular diseases related morbidity and mortality. Blood pressure is not well controlled in the majority of patients with both diabetes and hypertension. The main objective of this study was to assess blood pressure control and its determinants among diabetes mellitus co- morbid hypertensive ambulatory patients.

**Methods:**

Hospital based cross sectional study was conducted among diabetes mellitus co-morbid hypertensive ambulatory adult patients based on the inclusion criteria. Patient specific data was collected using structured data collection tool. Data was analyzed using statistical software package, SPSS version 20.0. To identify the independent predictors of blood pressure control, multiple stepwise backward logistic regression analysis was done. Statistical significance was considered at *p*-value <0.05. Patient’s written informed consent was obtained after explaining the purpose of the study. Patients were informed about confidentiality of the information obtained.

**Results:**

From a total of 131 study participants 51.14% were males with the mean (SD) age of the 50.69 ± 13.71. The mean duration of time since the diagnosis of hypertension was 7.44 ± 5.11 years. The mean (SD) SBP was 149.79 ± 16.32 mmHg, while the mean (SD) DBP was 89.77 ± 9.34 mmHg. More than one fourth (25.20%) of study participants had a controlled SBP, while about 27.48% had a controlled DBP. The overall control of BP was achieved in about 57 (43.51%) of the study participants. Older age (≥50 years) (AOR = 2.06; 95% CI: 2.65–7.79; *P* = 0.002), female gender (AOR = 1.42; 95% CI: 1.19–2.14; *P* = 0.042), duration of hypertension (AOR = 2.88, 95% CI: 1.27, 8.31, *P* = 0.02), non-adherence (AOR 2.05; 95% CI: 2.61–9.33; *P* = 0.01) and uncontrolled blood sugar(AOR = 1.65; 95% CI: 2.14–3.32; *P* = 0.04) are independent predictors for uncontrolled blood pressure.

**Conclusions:**

Blood pressure control to target goal was suboptimal in the study area. Diabetic patients who were older, female, live longer duration with hypertension, non-adherent to their medications and poor glycemic control were more likely to have uncontrolled BP. Therefore, more effort should be dedicated to control the blood pressure in diabetics.

## Background

Hypertension is the major contributor to the global burden of disease and to global mortality [1]. It is defined as persistently elevated arterial blood pressure (BP), systolic BP (SBP) ≥ 140 mmHg and/or diastolic BP (DBP) ≥ 90 mmHg [2, 3]. These numbers apply to all adults older than 18 years and indicate the level of BP at which the institution of therapy reduces hypertension related morbidity and mortality [3], although for patients aged 60 years or older a SBP up to 150 mmHg and a DBP of less than 90 mmHg is now regarded as acceptable [4]. The global prevalence of raised BP in adults aged 18 years and over was around 22% in 2014 [5] and the proportion is estimated to rise to over 29% by 2025 [6]. The prevalence of hypertension was highest in Africa, at 30% for all adults combined in 2014 [7]. Hypertension has shown a rapid increase in prevalence affecting significant numbers of individuals in Sub- Saharan Africa [8]. The prevalence in Sub- Saharan Africa is in the range of 25.4%- 41.1% in men and 27.2%- 38.7% in women [9]. The reported prevalence of hypertension in different regions of Ethiopia varied widely [10, 11]. The prevalence in the country is estimated to be between 20% and 30% [12]. Hypertension also clusters with other cardio-metabolic conditions, namely diabetes, dyslipidemia, insulin resistance, glucose intolerance and obesity [10], which together increase cardiovascular disease risk [10–12].

There are several co-existing factors in diabetic patient which contribute for and accelerate the progression of the atherosclerotic vascular complications. Hypertension, which is the most prevalent and independent cardiovascular risk factor in the general population, is extremely common problem in diabetics [4]. Diabetes approximately doubles risk of cardiovascular disease and concomitant hypertension nearly doubles that risk again. In addition to diabetes related renal dysfunction, hyperinsulinemia, extracellular fluid volume expansion, and increased arterial stiffness have been proposed as contributing factors for the development of hypertension in diabetics [3]. Based on the evidence provided by clinical trials, current guidelines recommend that patients who have been diagnosed with cardiovascular disease and its equivalents should reduce their blood pressure to <140/90 mmHg (so does in the case diabetics) [7]. Further, it is recommended that low density lipoprotein cholesterol (LDL-C) levels should be reduced to <100 mg/dL (2.5 mmol/L). Unfortunately, these therapeutic goals are not always attained [4, 7].

Drug treatments and life style interventions can be used for the management of hypertension [2]. Several lifestyle interventions have been shown to reduce BP. Apart from contributing to the treatment of hypertension; these strategies are beneficial in managing most of the other cardiovascular disease (CVD) risk factors [3]. Lifestyle modifications should be encouraged for all patients, regardless of stage of hypertension and includes smoking cessation, weight management, and reduction of dietary sodium intake, physical activity and moderation of alcohol consumption [13]. In general, lifestyle changes should be regarded as a complement to drug therapy rather than an alternative [7]. Drug treatment of hypertension depends on the degree of BP elevation and presence of compelling indications [2]. Most patients with stage 1 hypertension should be initially treated with a first-line antihypertensive drug, or a combination of two agents. Combination drug therapy is recommended for patients with stage 2 hypertension using preferably two first-line antihypertensive drugs [4]. According to the eighth report of the Joint National Committee (JNC 8); in the general non-black population, including those with diabetes, initial antihypertensive treatment should include a thiazide-type diuretic, calcium channel blockers (CCBs), angiotensin-converting enzyme inhibitors (ACEIs), or angiotensin receptor blockers (ARBs); whereas in the general black population, including those with diabetes, initial antihypertensive treatment should include a thiazide-type diuretic or CCB. For patients with chronic kidney disease (CKD), initial (or add-on) antihypertensive treatment should include an ACEI or ARB to improve kidney outcomes [4]. All the first line drugs classes have comparable outcome benefits [14].

The success of blood pressure management in hypertensive individuals is determined by the integrated use of optimal dose and regimen of anti-hypertensive medications together with an intervention targeted on life style modification. These have to be guided by the knowledge of the specific characteristics of patients group. Since the success of treating hypertension has been limited, and despite well-established approaches to diagnosis and treatment, in many communities fewer than half of all hypertensive patients have adequately controlled BP. This result will be expected to be lower among diabetic co-morbid patients. To date there is no single study done in Ethiopia to assess blood pressure control among hypertensive diabetics. Thus, this study is designed to highlight the status of uncontrolled hypertension in patients with type 2 diabetes and determine the associated factors, which may affect the blood pressure management and find the bottle neck and pave the way for interventions.

## Methods

### Study design and setting

A hospital based cross-sectional study design was conducted in March, 2017 at ambulatory clinic of Jimma University Medical Center (JUMC) among diabetes co-morbid hypertensive patients who fulfill the inclusion criteria. JUMC is the only teaching and referral hospital in the southwestern part of Ethiopia with bed capacity of 600. Geographically, it is located in Jimma town 352 km Southwest of Addis Ababa, the capital. It provides services for approximately 9000 inpatient and 80,000 outpatient clients per year with a catchment population of about 15 million people. All ambulatory hypertensive diabetic patients aged 18 years and older who were on antihypertensive treatment for at least 6 months before the study period and were on follow-up were included in the study.

### Sample size and sampling procedure

The sample size was determined by using the single population proportion formula, assuming a 95% confidence interval (CI), a prevalence of 50% for blood pressure control, a 5% margin of error, and a 10% non-response rate. The final sample size was 131 diabetic hypertensive patients. Random sampling technique was used to select 131 study participants after obtaining patient list from Chronic Illness Clinic Registration Book, which had lists of 557 hypertensive patients, of which 173 were with diabetes comorbidity.

### Data collection, procedure and quality control

A structured data collection questionnaire was developed by researchers from relevant literatures. Patient chart review and self-report was used to determine the various variables. Two trained data collectors interviewed the study participants and review patient charts and medical records for the respective information after all data collection tools are pre-tested. Before entry to SPSS for analysis, data was cleared, categorized, compiled and coded and also checked for completeness, accuracy. Any erroneous, ambiguous and incomplete data was excluded.

### Data processing and analysis

Data was entered into computer using EpiData version 3.1 and exported to the Statistical package for Social Science (SPSS) version 22.0 for analysis. Differences between mean values was evaluated using Student’s t test while proportions was compared using the Pearson’s Chi-square test. Multivariable logistic regression analyses were used to assess the crude and adjusted effect of seemingly significant predictors of the target outcome.

Categorical and continuous data was expressed as percentages and mean ± standard deviation respectively. Descriptive statistics was applied for the analysis of patient characteristics, including means, standard deviations (SD), medians, and percentiles and categorical variables was analyzed by using the chi-square test. A *p*-value of <0.05 was considered to be statistically significant.

### Ethical consideration

Ethical clearance & approval was obtained from institution review board (IRB) of Jimma University. The data that were collected from JUMC ambulatory clinic was preceded by a formal request letter from Jimma University. Written informed consent was taken from each study participant after clear orientation of the study objective. The raw data were not made available to any one and not used as the determinant of the participant. All steps in data collection and compilation were conducted and supervised by the researchers. Strict confidentiality was assured through anonymous recording and coding of questionnaires and placed in safe place. The patient got full right not to participate and as well as leave the study at any time during the study time.

### Operational definitions

- ■ Co-morbidity: Diseases or disorders that exist together with an index disease or co-occurrence of two or more diseases or disorders in an individual.
- ■ Controlled BP: BP < 140/90 mmHg in hypertensive diabetic patients of all ages [4].
- ■ Uncontrolled BP: BP ≥ 140/90 mmHg in hypertensive diabetic patients of all ages [4].
- ■ Adherent: a patient with a MMMAS score of ≥6 [15] .
- ■ Non adherent: a patient with a MMMAS scores of <6 [15].
- ■ Physically active: an individual who perform physical exercise for at least 30 min per day for at least 5 day per week.
- ■ Physically inactive: an individual who perform physical exercise for less than 30 min per day for less than 5 day per week [16].
- ■ Controlled blood sugar: Fasting blood sugar less than 130 mg/dL [17].
- ■ Uncontrolled blood sugar: Fasting blood sugar less than 130 mg/dL [17].

## Results

### Socio-demographic and behavioral characteristics

Overall, 131 participants were included in this study. Majority of study participants were males 67 (51.14%). The mean weight of the respondents was 67.9 ± 13.01. The mean age of the respondents was 50.69 ± 13.71 and majority of them 67 (51.14%) were age of ≥50 years, 109 (83.20%) were married. Among the participants, 21(16.03%) were current smokers and 23 participants reported their current use of alcohol and 35 (26.72%) had no formal education and 44 (33.60%) were unemployed. About three forth of the study participants were found to be adherent to their medication according to a self-reported measure of adherence using the eight item MMMAS (Table 1).

**Table 1:** Baseline socio-demographic characteristics of study participants at JUMC, 2017

Variables		Frequency	Percentage
Age(years)	18–35	18	13.74
	36–50	46	35.12
	≥ 50	67	51.14
Weight (kg)(Mean± SD)	67.9 ± 13.01		
Gender	Male	67	51.14
	Female	64	48.86
Marital Status	Single	11	8.40
	Married	109	83.20
	Divorced	4	3.05
	Widowed	7	5.35
Educational status	No formal education	35	26.72
	Primary school	33	25.19
	Secondary school	27	20.61
	College and above	36	27.48
Monthly Income (ETB)	No regular income	41	31.30
	<1000	21	16.03
	1000–2000	19	14.50
	2000–3000	18	13.74
	≥ 3000	32	24.43
Residence	Rural	40	30.53
	Urban	91	69.47
Job/occupation	Gov’t employee	30	22.90
	Non-Gov’t employee	19	14.50
	Self employed	38	29.00
	Unemployed	44	33.60
Living status	Living with immediate family	113	86.25
	Living with Extended family	10	7.63
	Living alone	5	3.82
	Other*	3	2.30
Smoker		21	16.03
Alcoholic		23	17.56
Chat chewer		36	27.48
Adherence level	Adherent	97	74.05
	Non-adherent	34	25.95

*Living in prison

### Clinical characteristics of participants

The clinical characteristics of patients showed that 23 (17.56%) of participants had a family history of hypertension and 18 (13.74%) had family history of diabetes. About 46.56% and 48.10% of patients were living with hypertension and diabetes for more than five [5] years, respectively. More than half the patients had a monthly follow up at JUMC ambulatory clinic. From 43 (32.82%) patients with co-morbid conditions, 21 (16.03%) had eye problem. The mean duration of time since the diagnosis of hypertension was 7.44 ± 5.11 with a range of 0.7–22 year. With regard to physical exercise; 56 (42.74%) of participants reported to perform physical exercise from whom 35(62.50%) were physically active. Among the study participants, 117 (89.31%) reduce salt in their food (Table 2).

**Table 2:** Baseline clinical characteristics of study participants at JUMC, 2017

Variables		Frequency	Percentage
Family history of hypertension	Yes	23	17.56
	No	108	82.44
Family history of Diabetes	Yes	18	13.74
	No	113	86.26
Time since hypertension diagnosis(years)	≤ 1	25	19.08
	2–5	45	34.35
	≥ 5	61	46.56
Time since diabetes diagnosis(years)	≤ 1	21	16.03
	2–5	47	35.87
	≥ 5	63	48.10
Frequency of followup(refill)	Monthly	71	54.20
	Every two months	41	31.30
	Every three months	19	14.50
Do physical activities	Yes	56	42.74
	No	75	57.26
Physical active	Yes	35	62.50
	No	21	37.50
Reduce salt intake	Yes	117	89.31
	No	14	10.69
Blood glucose	Controlled	86	65.65
	Uncontrolled	45	34.35
Comorbidity	Heart failure	5	3.82
	HIV/AIDS	2	1.53
	CKD	5	3.82
	Asthma	3	2.30
	Retinopathy	21	16.03
	Other^a^	7	5.34

^a^Epilepsy, peripheral neuropathy

### Antihypertensive and hypoglycemic medications

The overall utilization of antihypertensive drugs showed that, majority of patients were on dual antihypertensive (67.94%**).** About one thirds of the patients were on combination of enalapril and amlodipine followed by hydrochlorothiazide and enalapril (17.55%) combinations. About 8.40% of patients were taking triple antihypertensive medications. About two thirds of the patients were taking insulin (Table 3).

**Table 3:** Antihypertensive & hypoglycemic Agents among study participants at JUMC, 2017

Medications	Frequency	Percentage
1. Antihypertensive agents		
Monotherapy	31	23.66
Enalapril	14	10.68
Amlodipine	9	6.87
Nifedipine	4	3.05
Hydrochlorothiazide	3	2.29
Atenolol	1	0.76
Dual therapy	89	67.94
Hydrochlorothiazide + Enalapril	23	17.55
Hydrochlorothiazide + Atenolol	7	5.34
Enalapril + Amlodipine	43	32.82
Atenolol + Amlodipine	2	1.53
Enalapril + Atenolol	12	9.16
Losartan +Amlodipine	2	1.53
Triple Therapy	11	8.40
Hydrochlorothiazide + Enalapril + Amlodipine	5	3.82
Furosemide + Enalapril +Amlodipine	2	1.53
Metoprolol + Enalapril + Furosemide	4	3.05
2. Antidiabetic agents		
Insulin	59	45.04
Oral hypoglycemic	45	34.35
Insulin + Oral hypoglycemic	27	20.61

### Blood pressure control among hypertensive diabetics

**Fig. 1:**
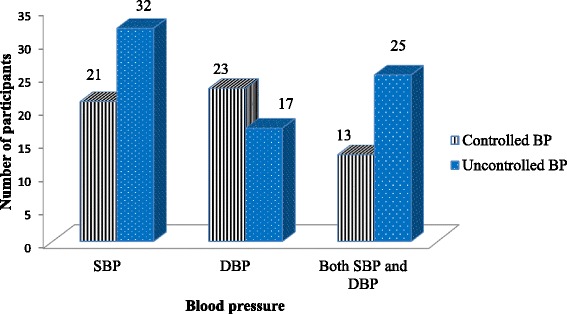
Blood pressure control among study participants at JUMC, 2017

The mean SBP was 149.79 ± 16.32, while the mean DBP was 89.7 ± 9.34. More than one fourth of study participants had a controlled SBP; while about 27.48% had a controlled DBP. The overall control of BP was achieved in about 57 (43.51%) of the study participants (Fig. 1).

### Determinants of uncontrolled blood pressure in diabetic patients

The association of independent variables with the dependent variable was investigated using both univariate and multivariate logistic regression techniques. In univariate logistic regression analysis; age, gender, marital status, education level, alcohol intake, smoking, duration with hypertension (HTN), frequency of follow up, blood sugar(glycemic control), comorbidities and medication adherence showed association with BP control and hence, were used in multivariate analysis. The result of the multivariate analysis showed age to be significantly associated with uncontrolled BP in that patients aged ≥50 years were two times (AOR = 2.06; 95% CI: 2.65–7.79; *P* = 0.002) more likely to have uncontrolled BP than those <50 years. Patients with tertiary level education were 74% (AOR = 0.26; 95% CI: 0.13–0.54; *P* = 0.03) less likely to have uncontrolled BP than those with no formal education. Gender was also found to have association with uncontrolled BP, in that female patients have 1.42 times more likely (AOR = 1.42; 95% CI: 1.19–2.14; *P* = 0.042). Duration of hypertension since diagnosis was also associated with uncontrolled BP, in that patients with a diagnosis longer than 5 years were almost three times (AOR = 2.88; 95% CI: 1.27–8.31; *P* = 0.02) more likely to have uncontrolled BP than those with a diagnosis of <5 years. Patients with a monthly BP measurement were 35% less likely to have uncontrolled BP than those with every three months BP measurement (AOR 0.65; 95% CI: 0. 63–0.98; *P* = 0.04) and non-adherent patients were two times more likely to have uncontrolled BP than adherents (AOR 2.05; 95% CI: 2.61–9.33; *P* = 0.01). Patients with uncontrolled blood sugar (poor glycemic control) were almost two times (AOR = 1. 65; 95% CI: 2.14–3.32; P = 0.04) more likely to have uncontrolled BP than with controlled blood sugar (Table 4).

**Table 4:** Determinants of uncontrolled blood pressure among study participants at JUMC, 2017

Variable		Blood Pressure		COR (95 %CI)	AOR (95 %CI)
		Controlled	Uncontrolled		
Age category	<50 years	20	44	1.00	1.00
	≥ 50 years	37	30	2.55 (1.72, 4.75)*	2.06 (2.65, 7.79)*
Gender	Male	33	34	1.00	1.00
	Female	24	40	1. 63 (0. 98, 2. 90)*	1.42 (1.19, 2.14)*
Marital status	Married	45	64	1.00	1.00
	Single	7	4	2.721 (10.59, 13.36)	3.42 (11.22, 14.12)
	Divorced	2	2	0.55 (0.14, 5.53)	0.49 (0.54, 1. 59)
	Widowed	3	4	1.46 (0.22, 0.78)*	1.76 (0.46, 1.27)
Education level	No formal education	7	28	1.00	1.00
	Primary	12	21	2.22 (1.80, 2.84)	0. 18 (0. 15, 1. 13)
	Secondary	15	12	1. 15 (0. 98, 2. 35)	0. 07 (0.37, 1. 53)
	Tertiary and above	23	13	0. 27 (0. 34, 1. 12)*	0. 62 (0. 31, 0. 45)*
Residence	Rural	18	22	1.00	1.00
	Urban	39	52	0. 28 (1. 34, 11. 75)	2.11 (2.47, 9.16)
Monthly income (ETB)	No regular income	15	26	2.17 (4.42, 6.15)	1.23 (0.23, 4.74)
	<1000	6	15	1.06 (1.24, 3.1 5)	1.09 (2.12, 6.11)
	1000–2000	9	10	0.69 (1.79, 3.35)	0.453 (0.32, 7.42)
	2000–3000	7	11	0.66 (0. 64, 5. 59)	0.23 (2.32, 9.47)
	≥ 3000	20	12	1.00	1.00
Occupation	Gov’t employee	21	9	1.00	1.00
	Non-Gov’t employee	13	6	0. 28 (0. 34, 1. 75)	1. 10 (0. 74, 2. 61)
	Self employed	9	29	0.67 (0.27, 1.66)	0.68 (0.25, 1.87)
	Unemployed	14	30	1.06 (0.60, 1.85)	1.05 (0.57, 1.94)
Smoking	No	52	58	1.00	1.00
	Yes	5	16	1.31 (0.75, 2.28)*	1.14 (0.62, 2.10)
Alcohol intake	No	53	55	1.00	1.00
	Yes	4	19	1.66 (0.46, 5.95)*	1.23 (0.32, 4.74)
Chat chewer	No	42	53	1.00	1.00
	Yes	15	21	1.05 (0.67, 1.66)	1.12 (0.68, 1.84)
Adherence	Adherent	49	48	1.00	1.00
	Non-adherent	8	26	2. 37 (2.71, 8. 65)*	2.05 (2.61, 9.33)*
Frequency of follow up	Monthly	40	31	0. 35 (0. 53, 0. 97)*	0. 65 (0. 63, 0. 98)*
	Every 2 months	10	31	1.31 (0.75, 2.28)	1. 4 2 (2. 26, 3.01)
	Every 3 months	7	12	1.00	1.00
Duration with Hypertension	≤ 1 year	13	12	1.00	1.00
	2–5 years	20	25	0. 69 (1. 36, 11.44)	1.11 (2.62, 6.63)
	≥ 5 years	24	37	1. 75 (1. 59, 5. 85)*	2.88 (1.27, 8.31)*
Blood Sugar	Controlled	51	35	1.00	1.00
	Uncontrolled	6	39	2.33 (2. 11, 7. 15)*	1. 65 (2. 14,3. 32)*
Comorbidity	No	40	48	1.00	1.00
	Yes	17	26	1.04 (0. 44, 3. 31)*	1.12 (1. 22, 4. 45)
Anti-HTN regimen	Monotherapy	13	18	1.00	1.00
	Dual Therapy	40	49	048 (0.13, 1.08)	0.44 (1.10, 2.11)
	Triple therapy	4	7	1.35 (1.79, 4.78)	1.68 (1.19, 3.43)

*ETB* Ethiopian Birr, *Anti-HTN* Antihypertensive

*P-value less than 0.05

## Discussion

Hypertension in patients with diabetes is a well-recognized cardiovascular risk factor [18]. Recently the 8th Report of the Joint National Committee on prevention, detection, evaluation and treatment of high blood pressure (JNC 8) concluded that BP measurement in diabetic patients should be 140/90 mmHg or less [4]. Hypertension is twice as common in persons with diabetes as it is in others. Hypertension is known to contribute to diabetic micro-and macro-vascular complications [19]. To reduce the risk, hypertension must be diagnosed accurately and promptly, and the patient must receive adequate treatment. However, new guidelines have been published to stress on the importance of aggressive blood pressure control in diabetic [2]. This study attempted to evaluate the blood pressure control among diabetic patients in Jimma University medical center.

The result of the study showed that from a total of 131hypertensive diabetics only 43.51% of the patients met the currently recommended BP for diabetes of <140/90 mmHg [4]. The level of BP control found in this study is lower than studies from Chilean (59.7%) [20], Greece (55.6%) [19], USA (49.8%) [21] and South Africa (57%) [22–24]. It was also lower as compared study done by Greenberg et al. [14] and Berlowitz et al. [25]. But, it comparable with based studies from Adama (43.6%) [26] and Nigeria (42%) [27], respectively. This difference in the level of BP control might be due to a diabetes comorbidity of our study population, which may indicate the need of more effort to control BP in diabetics.

This study revealed that 33 (25.20%) and 36 (27.48%) of the study participants had a controlled SBP and DBP respectively, which is lower than a report from Saudi Arabia (40.4% and 51.6%) [28] and USA (55.7% and 77.1%) [29].This difference in the level of control of SBP and DBP might be due to age related increase in SBP as more than half of study participants were older than 50 years of age.

As from this report age was significantly associated with uncontrolled BP in that patients aged greater 50 years were two times more likely to have uncontrolled BP than those younger than 50 years (AOR = 2.06, 95% CI: 2.65, 7.79, *P* = 0.002). It is similar to study done by in USA [29] and Kenya [30]. As well similar to report from Brazil [31], this study showed older age as contributing factor for uncontrolled BP. In fact, an interaction between biological and behavioral factors could be used to explain our results. Regarding biological factors, a possible explanation would be the natural processes related to ageing, such as autonomous imbalance and vessels stiffening. In relation to behavioral factors, previous studies showed that older people have decreased physical activity practice [32].

Educational status was also found to be associated with BP control in which patients with tertiary education were 38% (AOR = 0.26, 95% CI: 0.13, 0.54, *P* = 0.03) less likely to have uncontrolled BP when compared to those with no formal education. This result is consistent with a result obtained from Chilean HC study which showed low education level to have a negative association with BP control [20]. This may be is a result of increased awareness regarding the treatment of hypertension, adherence to life style modifications to decrease BP or adherence to antihypertensive drug treatment. The result of the study showed evidence of association of frequent BP measurement with good odds of BP control in which patients with a monthly BP measurement were 35% less likely to have uncontrolled BP than those with a monthly BP measurement (AOR 0.65, 95% CI: 0. 63, 0. 98, *P* = 0.04)). This may be is a result of a health seeking behavior, frequent adjustment in life style related factors and a tendency to adhere to antihypertensive medication among those who frequently measure their BP.

Adherence to therapies is a primary determinant of treatment success. Poor adherence attenuates optimum clinical benefits and therefore reduces the overall effectiveness of treatment outcome this study showed that non adherent patients were two times more likely to have uncontrolled BP than adherents (AOR 2.05, 95% CI: 2.61, 9.33, *P* = 0.01). This result is consistent with the result obtained from studies from South Africa [24], Zimbabwe [33], USA [34] and Nigeria [35].

In light of this result, patients should be counseled and encouraged to adhere to antihypertensive medications, as adherence to antihypertensive medications is a key to achieving an optimal BP, specially, in patient needing intensive BP control, such as comorbid diabetics. Those patients with positive behavioral measures (i.e. smoking, alcohol intake and chat chewing) had higher odds of uncontrolled BP, which is consistent with study done in Saudi Arabia (8.3%) [28], Oman (8%) [36] and Zimbabwe (16.1%) [33]. This could be secondary to the oxidative stress they impose on the cell as well as their effect on adherence [37]. Thus, cessation of cigarette smoking and decreasing/stopping alcohol intake is recommended to achieve an optimal BP among patients with hypertension, specifically comorbid with diabetes. Because, this populations have additional cardiovascular risk equivalent.

This study also reveals that, not only comorbidity with diabetes, but also poor glycemic control is an independent predictor for uncontrolled BP. Patients with poor glycemic control had almost two times (AOR = 1. 65, 95% CI: 2. 14, 3. 32, *P* = 0.04) more likely to develop uncontrolled BP. This study is consistent with study done in Brazil [31], by Greenberg et al. [14], Berlowitz et al. [25], Adler et al. [38] and Jansson et al. [17]. This could be explained by the pathophysiologic process related specifically to the presence of diabetes may involve excess circulating insulin. Excess circulating insulin, arising from insulin resistance in diabetes, may increase blood pressure by stimulating the sympathetic nervous system, acting as a growth factor, and/or increasing sodium reabsorption in the kidneys [39]. Simply, the release of insulin following a meal stimulates vasodilation (the widening of blood vessels) in skeletal muscle while also activating the sympathetic nervous causing vasoconstriction [40].

## Conclusions

Blood pressure control to target goal was suboptimal and achieved only in almost two fifth of pharmacologically treated hypertensive diabetic patients attending ambulatory clinic of Jimma University Medical Center. Diabetic patients who were older, female, live long duration with hypertension were more likely to have uncontrolled BP. These patients can be clearly identified and therefore preventive measures should concentrate on this group of patients. Older age, female gender, duration of hypertension, non-adherence and uncontrolled blood sugar are independent predictors for uncontrolled blood pressure among hypertensive diabetic patients. Monthly follow up and higher education are protective for blood pressure control.

## References

[1] Organization WH (2013). A global brief on hypertension: silent killer, global public health crisis: world health day 2013.

[2] Chobanian AV, Bakris GL, Black HR, Cushman WC, Green LA, Izzo JL (2003). Seventh report of the joint national committee on prevention, detection, evaluation, and treatment of high blood pressure. Hypertension.

[3] Longo DL, Fauci AS, Kasper DL, Hauser SL, Jameson JL, Loscalzo J. Harrison’s Principles of Internal Medicine 18E Vol 2 EB. McGraw Hill Professional. 2012. p. 1611–1624.

[4] James PA, Oparil S, Carter BL, Cushman WC, Dennison-Himmelfarb C, Handler J (2014). 2014 evidence-based guideline for the management of high blood pressure in adults: report from the panel members appointed to the eighth joint National Committee (JNC 8). JAMA.

[5] Organization WH (2014). Noncommunicable diseases country profiles 2014.

[6] Kuller LH (2007). Epidemic hypertension in sub-Saharan Africa. Hypertension.

[7] Kearney PM, Whelton M, Reynolds K, Muntner P, Whelton PK, He J (2005). Global burden of hypertension: analysis of worldwide data. Lancet.

[8] Addo J, Smeeth L, Leon DA (2007). Hypertension in sub-Saharan Africa. Hypertension.

[9] Awoke A, Awoke T, Alemu S, Megabiaw B (2012). Prevalence and associated factors of hypertension among adults in Gondar, Northwest Ethiopia: a community based cross-sectional study. BMC Cardiovasc Disord.

[10] Tesfaye F, Byass P, Wall S (2009). Population based prevalence of high blood pressure among adults in Addis Ababa: uncovering a silent epidemic. BMC Cardiovasc Disord.

[11] Molla M (2015). Systematic reviews of prevalence and associated factors of hypertension in Ethiopia: finding the evidence. Sci J Public Health.

[12] Kibret KT, Mesfin YM (2015). Prevalence of hypertension in Ethiopia: a systematic meta-analysis. Public Health Rev.

[13] Mancia G, Fagard R, Narkiewicz K, Redon J, Zanchetti A, Böhm M (2013). 2013 ESH/ESC guidelines for the Management of Arterial Hypertension: the task force for the management of arterial hypertension of the European Society of Hypertension (ESH) and of the European Society of Cardiology (ESC). Blood Press.

[14] Greenberg JD, Tiwari A, Rajan M, Miller D, Natarajan S, Pogach L (2006). Determinants of sustained uncontrolled blood pressure in a national cohort of persons with diabetes. Am J Hypertens.

[15] Morisky DE, Ang A, Krousel-Wood M, Ward HJ (2008). Predictive validity of a medication adherence measure in an outpatient setting. The. J Clin Hypertens.

[16] Haskell WL, Lee I-M, Pate RR, Powell KE, Blair SN, Franklin BA (2007). Physical activity and public health: updated recommendation for adults from the American College of Sports Medicine and the American Heart Association. Circulation.

[17] Jansson S, Svärdsudd K, Andersson D (2014). Effects of fasting blood glucose levels and blood pressure and treatment of diabetes and hypertension on the incidence of cardiovascular disease: a study of 740 patients with incident type 2 diabetes with up to 30 years’ follow-up. Diabet Med.

[18] Turner R, Millns H, Neil H, Stratton I, Manley S, Matthews D (1998). Risk factors for coronary artery disease in non-insulin dependent diabetes mellitus: United Kingdom prospective diabetes study (UKPDS: 23). BMJ.

[19] Skliros E, Vasibossis A, Loumakis P, Sotiropoulos A, Giannakaki G, Razis N (2003). Evaluation of hypertension control in Greek primary care units. The VANK study. J Hum Hypertens.

[20] Sandoval D, Bravo M, Koch E, Gatica S, Ahlers I, Henríquez O (2012). Overcoming barriers in the management of hypertension: the experience of the cardiovascular health program in Chilean primary health care centers. Int J Hypertens.

[21] Shelley D, Tseng T-Y, Andrews H, Ravenell J, Wu D, Ferrari P (2011). Predictors of blood pressure control among hypertensives in community health centers. Am J Hypertens.

[22] Adeniyi OV, Yogeswaran P, Longo-Mbenza B, Ter Goon D (2016). Uncontrolled hypertension and its determinants in patients with concomitant type 2 diabetes mellitus (T2DM) in rural South Africa. PLoS One.

[23] Choukem SP, Kengne AP, Dehayem YM, Simo NL, Mbanya JC (2007). Hypertension in people with diabetes in sub-Saharan Africa: revealing the hidden face of the iceberg. Diabetes Res Clin Pract.

[24] Onwukwe SC, Omole OB (2012). Drug therapy, lifestyle modification and blood pressure control in a primary care facility, south of Johannesburg, South Africa: an audit of hypertension management. South African. Fam Pract.

[25] Berlowitz DR, Ash AS, Hickey EC, Glickman M, Friedman R, Kader B (2003). Hypertension management in patients with diabetes. Diabetes Care.

[26] Hussein M, Lenjisa J, Woldu M, Tegegne G, Umeta G (2014). Assessment of drug related problems among hypertensive patients on follow up in Adama hospital medical college, East Ethiopia. Clinic Pharmacol Biopharmaceut.

[27] Adebolu FA, Naidoo M (2014). Blood pressure control amongst patients living with hypertension presenting to an urban district hospital outpatient clinic in Kwazulu-Natal. Afr J Prim Health Care Fam Med.

[28] Al-Tuwijri AA, Al-Rukban MO (2006). Hypertension control and co-morbidities in primary health care centers in Riyadh. Ann Saudi Med.

[29] Ornstein SM, Nietert PJ, Dickerson LM (2004). Hypertension management and control in primary care: a study of 20 practices in 14 states. Pharmacotherapy.

[30] Mutua EM, Gitonga MM, Mbuthia B, Muiruri N, Cheptum JJ, Maingi T (2014). Level of blood pressure control among hypertensive patients on follow-up in a regional referral hospital in Central Kenya. Pan Afr Med J.

[31] Codogno JS, Fernandes RA, Freitas Jr IF, Monteiro HL. Determinants of blood pressure in type 2 diabetic subjects with high occurrence of inadequate glycemic control. Medicina (Brazil). 2012;45(1):49–57.

[32] Wichi RB, De Angelis K, Jones L, Irigoyen MC (2009). A brief review of chronic exercise intervention to prevent autonomic nervous system changes during the aging process. Clinics.

[33] Goverwa TP, Masuka N, Tshimanga M, Gombe NT, Takundwa L, Bangure D (2014). Uncontrolled hypertension among hypertensive patients on treatment in Lupane District, Zimbabwe, 2012. BMC Res Notes.

[34] Elperin DT, Pelter MA, Deamer RL, Burchette RJ (2014). A large cohort study evaluating risk factors associated with uncontrolled hypertension. J Clin Hypertens.

[35] Iloh GU, Ofoedu JN, Njoku PU, Godswill-Uko EU, Amadi AN (2013). Medication adherence and blood pressure control amongst adults with primary hypertension attending a tertiary hospital primary care clinic in eastern Nigeria. Afr J Prim Health Care Fam Med.

[36] Al-Saadi R, Al-Shukaili S, Al-Mahrazi S, Al-Busaidi Z (2011). Prevalence of uncontrolled hypertension in primary care settings in al seeb wilayat, oman. Sultan Qaboos Univ Med J.

[37] Davis TM, Millns H, Stratton IM, Holman RR, Turner RC (1999). Risk factors for stroke in type 2 diabetes mellitus: United Kingdom prospective diabetes study (UKPDS) 29. Arch Intern Med.

[38] Adler AI, Stratton IM, Neil HAW, Yudkin JS, Matthews DR, Cull CA (2000). Association of systolic blood pressure with macrovascular and microvascular complications of type 2 diabetes (UKPDS 36): prospective observational study. BMJ.

[39] Singh M, Mensah GA, Bakris G (2010). Pathogenesis and clinical physiology of hypertension. Cardiol Clin.

[40] Muniyappa R, Montagnani M, Koh KK, Quon MJ (2007). Cardiovascular actions of insulin. Endocr Rev.

